# Economic Evaluation of Combination Therapy Versus Monotherapy for Treatment of Benign Prostatic Hyperplasia in Hong Kong

**DOI:** 10.3389/fphar.2018.01078

**Published:** 2018-10-16

**Authors:** David Bin-Chia Wu, Chi Hang Yee, Chi-Fai Ng, Shaun Wen Huey Lee, Nathorn Chaiyakunapruk, Yu-Shan Chang, Kenneth Kwing Chin Lee

**Affiliations:** ^1^School of Pharmacy, Monash University Malaysia, Bandar Sunway, Malaysia; ^2^Asian Centre for Evidence Synthesis in Population, Implementation and Clinical Outcomes, Health and Well-being Cluster, Global Asia in the 21st Century (GA21) Platform, Monash University Malaysia, Bandar Sunway, Malaysia; ^3^Division of Urology, Department of Surgery, The Chinese University of Hong Kong, Shatin, Hong Kong; ^4^School of Pharmacy, University of Wisconsin, Madison, WI, United States; ^5^Department of Pharmacy Practice, Faculty of Pharmaceutical Sciences, Center of Pharmaceutical Outcomes Research, Naresuan University, Phitsanulok, Thailand; ^6^College of Commerce, Tamkang University, New Taipei City, Taiwan

**Keywords:** benign prostatitc hypertrophy, cost effectiveness, dutasteride, economic, combination therapy

## Abstract

**Background:** Lower urinary tract symptoms (LUTS) suggestive of benign prostatic hyperplasia (BPH) is a common condition affecting men. Studies have shown that the prevalence of LUTS/BPH increases with age, which will cause considerable economic burden to the healthcare system and society. The aim of the present study was to evaluate the long term cost effectiveness of dutasteride and tamsulosin therapy compared to tamsulosin alone in men with BPH in Hong Kong.

**Methods:** A Markov decision model was constructed to estimate the economic impact from a healthcare payers’ perspective, which only included direct costs. Analyses were conducted for a 4-year time frame.

**Results:** When compared to tamsulosin alone, combination therapy was more expensive but also more effective in preventing complications and reduced the need for surgery. Over life-time projection suggest that combination therapy will be cost-effective if the willingness-to pay threshold of USD 20,000.

**Conclusion:** Findings of this study found that combination therapy of tamsulosin and dutasteride was more cost-effective compared to tamsulosin alone across a wide range of scenario.

## Introduction

Lower urinary tract symptom (LUTS) suggestive of benign prostatic hyperplasia (BPH) is a common condition among middle-aged to elderly men. It is characterized by a cluster of chronic urinary symptoms in the bladder, prostate, and a major cause of benign prostatic hyperplasia. Population based studies suggested that nearly one in every four men aged 50 and above is affected by LUTS (Lee et al., 2017). Similarly, a recent study found that 69.3% of community-dwelling men experienced moderate-to-severe symptoms on International Prostate Symptom Score (IPSS) in Hong Kong (Yee et al., 2014). Without proper treatment, LUTS can present with complications, such as acute urinary retention (AUR), urinary tract infections, or sometimes obstructive uropathy (Lee et al., 2007;McVary et al., 2011). In the current era of an increased life expectancy and the aging of baby boomer generation, male LUTS would become an issue of increasing socioeconomic and medical importance (Lee et al., 2008; Kaplan et al., 2015; Lee et al., 2017).

The main pharmacological agents for the management of LUTS are alpha-blockers and 5-alpha-reductase inhibitors (5ARIs). Currently, international guidelines (McVary et al., 2011; Oelke et al., 2013) recommend the use of alpha-blockers for symptomatic relief in LUTS patients who do not have a markedly enlarged prostate, while highlighting that these agents do not alter the natural progression of the disease. On the other hand, 5ARIs, which can be administered as monotherapy or in combination with alpha-blockers, are recommended for symptomatic men with an enlarged prostate and are associated with decreased risk of urinary retention and related surgery. One of the major trials to support such recommendation is the Combination of Avodart^TM^ (dutasteride) and tamsulosin (CombAT) trial (Roehrborn et al., 2010, 2011). In this randomized- multicenter, double-blind, parallel-group study of 50 years or older men with a clinical diagnosis of moderate to severe BPH, a single-dose tamsulosin/dutasteride combination therapy was compared with tamsulosin and dutasteride monotherapies. It was found that patients with a prostate volume ≥40 ml had a lower risk of disease progression, AUR, and BPH-related surgery in the groups receiving dutasteride or combination therapies than in the group receiving tamsulosin monotherapy.

In terms of economic burden, BPH/LUTS is associated with high personal and societal costs, which are evident in direct medical costs and indirect losses in daily functioning, and through its negative impact on quality of life (QoL) for both patients and partners (Lee et al., 2008; Speakman et al., 2015). Treatment and interventions for BPH/LUTS are essential, which aim at providing symptom relief as well as addressing the root cause, while limiting the occurrence of adverse events. As the prevalence of BPH/LUTS increases with age, the burden on the healthcare system and society may increase due to the aging population (Rosen et al., 2003; Lee et al., 2017). In the United Kingdom, it was estimated that more than $180 million was spent on BPH treatments each year (Speakman et al., 2015). In 2000, it was estimated that the direct cost of medical services for BPH in the United Sates was about US$1.1 billion (Wei et al., 2005). In Hong Kong, the per capita total expenditure on health in 2010/2011 was HK$ 13302 (US$ 1705) (Department of Health, 2017). Like many places around the world, the healthcare systems local and abroad face an increasing cost scrutiny. Cost-effectiveness assessment of treatments is gaining more importance to ensure patient satisfaction as well as the most efficient outcome in the environment of cost-containment.

In view of this, the objective of our study was to evaluate the long-term cost-effectiveness of oral, daily, single-dose combination therapy dutasteride/tamsulosin (Duodart^®^), compared with oral, daily tamsulosin 0.4 mg in Hong Kong (HK).

## Materials and Methods

### Overview of the Economic Model

A Markov model was developed to project the overall cost-savings of combination therapy in a hypothetical cohort of 10,000 patients treated in the public healthcare sector of HK. As such, ethical approval was not obtained since no patient was involved in this research. The natural history of BPH progression has been widely examined in literature (Barry, 1990; Wei et al., 2005). As such, we used this as our base case to build our model. We subsequently had a discussion workshop to seek clinical experts’ views on the disease progression pathway of BPH and to understand the use of different treatments in the local context. The workshop included review of international BPH management guidelines, exploration of consensus in the local clinical setting, as well as retrospective analysis of BPH outcome data from the territory. The model population is consistent with the entry criteria for the CombAT trial (Roehrborn et al., 2010), i.e., the hypothetical cohort of patients ≥50 year of age with a BPH clinical diagnosis by medical history and physical examination. This age group was representative of BPH patient population in Asia and the most commonly studied age group in clinical trials.

**FIGURE 1:**
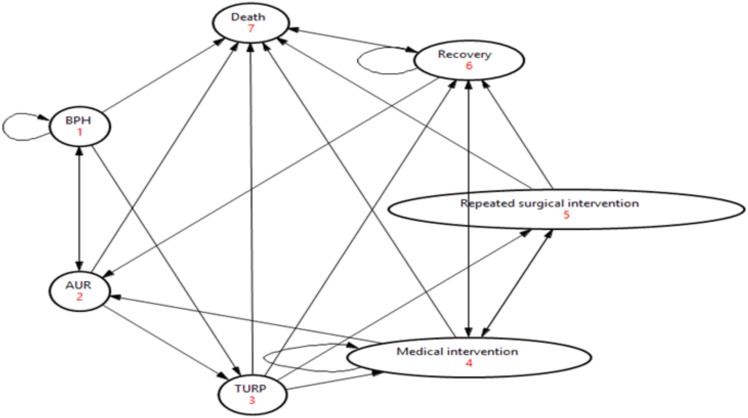
Model structure. ^$^The death state includes patients who died from TURP and all-cause mortality.

The model was structured according to the HK-specific treatment practice, and is shown in **Figure 1**. In the beginning of the simulation, patients start from BPH state. As time progresses, patients might remain in BPH state, transition between the symptom states, experience AUR, receive a surgical intervention (TURP), or die due to natural causes. After experiencing an AUR episode, the patient could transition back to BPH state. After TURP, a patient would receive medical intervention or have a repeated TURP procedure if the initial surgical procedure is not successful. Patients who have undergone TURP could die due to complications of the procedure, or they could die due to other causes. A patient in TURP, repeated TURP or medical intervention state could recover back to healthy state.

The analysis was performed from the healthcare payers’ perspective, which only included direct costs. A cycle length of 1 year was adopted to capture the full effect of both intervention in terms of resource use and quality of life. In the base-case scenario, the model was simulated over 4 years, i.e., duration of the trial. To assess the sensitivity of the model results to the time horizon, the model was also run for 35 years, i.e., patients’ life span. A discount rate of 3% was applied to both cost and outcome.

### Assumptions

There are a few assumptions made in our model as described in the following:

(1) In reality, BPH state can be classified according to severity, i.e., mild, moderate, and severe states. However, due to the lack of granular data on symptom severity as measured by the International Prostate Symptom Score (IPSS) of BPH patients, we modeled patients into one BPH state;
(2) It is assumed that high proportion of acute urinary retention (AUR) patients (80%) will receive transurethral resection of the prostate (TURP) procedure. This uncertainty will be addressed using 1-way and multivariate probabilistic sensitivity analyses;
(3) Patients who recover from the first TURP procedure are assumed to incur only one follow-up cost. Medical intervention means the use of pharmacological agents for follow-up management;
(4) Post-TURP death due to TURP-related complications is defined as death which occurs within 30 days after TURP procedure;
(5) The second TURP procedure will require different amount of resources compared to the first TURP; Based on local literature, post-TURP death due to second TUPR is assumed to be the same as that from first TURP;
(6) It is assumed that patients will receive at most 2 TURP procedures.

### Data Inputs

As summarized in **Table 1**, model parameters including transition probabilities, efficacy, costs, and utilities were derived from HK-specific sources, literatures and assumptions based on clinical inputs.

**Table 1:** Model inputs.

Parameters	Base-case	Range	Source
**Transition probability (monthly)**			
Remaining in BPH	0.345	0.248–0.45	Ball et al., 1981
BPH patients who experience TURP	0.021	0.0098–0.036	Ball et al., 1981
AUR patients going through TURP	0.8	0.75–0.85	Prince of Wales Hospital^∧^
TURP patients’ 30-day mortality	0.0237	0.0179–0.0296	Madersbacher and Marberger, 1999
TURP patients who recover	1	N/A	Madersbacher and Marberger, 1999
TURP patients who require medical intervention	0.059	N/A	Prince of Wales Hospital^%^
TURP patients who require repeated surgical intervention	0.059	N/A	Prince of Wales Hospital^%^
Patients with repeated TURP died from surgical procedure	0	N/A	Prince of Wales Hospital^%^
Patients who remain at recovery state	0.329	0.275–0.41	Prince of Wales Hospital
Recovered patients who require medical intervention	0.0467	0.0295–0.0744	Prince of Wales Hospital
Patients on medication who require the second TURP	0.036	0.0215–0.061	Prince of Wales Hospital
Patients with repeated TURP requiring medication intervention	0.0025	N/A	Prince of Wales Hospital^%^
Patients with repeated TURP who recover	1	N/A	Prince of Wales Hospital^%^
Patients remain on medication	0.393	0.378–0.438	Prince of Wales Hospital^%^
TURP patients who fully recover	0.998	0.978–1	Prince of Wales Hospital^%^
Patients on medication who transition to AUR state	0		Ball et al., 1981
BPH patients who experience AUR	0.004	0.00064–0.0154	Ball et al., 1981
All-cause mortality			HK life table
**Efficacy (over 4 years)**			
Efficacy of tamsulosin/dutasteride against AUR (vs. monotherapy)	0.676	0.527–0.778	Roehrborn et al., 2010
Efficacy of tamsulosin/dutasteride against TURP (vs. monotherapy)	0.706	0.577–0.795	Roehrborn et al., 2010
**Cost (per annum) (2018 US$)**^@^			
tamsulosin/dutasteride	464.97	348–581	Hong Kong public hospital formulary estimation
Tamsulosin	55.79	41.8–69.7	Hong Kong public hospital formulary estimation
Managing a patient who is initially in BPH moderate state	471	223–720	Prince of Wales Hospital^%^
Managing a patient who experiences an episode of AUR	1312	590–4,199	Prince of Wales Hospital^%^
TURP procedure	6334	4,549–8,119	Prince of Wales Hospital^%^
Managing a patient who requires medical intervention	371	123–620	Prince of Wales Hospital^%^
**Utility**			
BPH mild	0.993	0.94–1	Baladi et al., 1996
BPH moderate	0.903	0.86–0.95	Baladi et al., 1996
BPH severe	0.79	0.75–0.83	Baladi et al., 1996
BPH (weighted) (used in the model)^$^	0.876	0.83–0.92	Baladi et al., 1996
AUR	0.25	0.24–0.26	Cher et al., 1997
TURP^#^	0.25	0.24–0.26	Cher et al., 1997
Medical intervention	0.25	0.24–0.26	Assumption
Recovery	1.0	0.95–1	Assumption

*N//A, Not available, ^@^1US$ = 7.85HK$, ^∧^Based on 200 patients, ^%^Based on 109 patients, ^&^Tamsulosin/dutasteride cost price: US$1.274/tablet; Tamsulosin cost price: US$0.15/tablet, ^$^The model used weighted utility calculated based on the distribution of BPH severity in HK population, i.e., BPH mild (20%), BPH moderate (40%), and BPH severe (40%), ^#^The utility of a patient undergoing the second TURP is considered the same as that of the first TURP*.

The majority of the transition probabilities were derived from a cohort of 200 BPH patients from Prince of Wales Hospital. Those patients comprised of roughly 10% of overall BPH patients in HK thus were considered representative of all BPH patients’ profile in HK. The remaining probabilities were obtained from published literature where available. In addition to mortality due to TURP procedure, the model also allowed for patients to die from all-cause mortality which was obtained from HK life table. The efficacy of the different interventions were derived from a 4-years, multicentre, randomized, double-blind, parallel-group CombaT clinical trial which included 4,844 men ≥50 years of age with a clinical diagnosis of BPH, International Prostate Symptom Score ≥12 (Roehrborn et al., 2010). The acquisition cost price of combination therapy and tamsulosin were US$1.274 and US$0.15 per tablet respectively based on the acquisition cost of Prince of Wales Hospital, which is a public hospital. The costs are relatively similar across all public hospitals in Hong Kong, which account for the care of majority of patients with BPH in the territory. Similarly, the costs of management of BPH, AUR, TURP, and medical intervention were all solicited from Prince of Wales Hospital. Quality of life (utility) values associated with each health state were extrapolated from two other BPH cost-effectiveness studies (Baladi et al., 1996; Cher et al., 1997) as local utility study was not available. In the model, QALYs were calculated by applying the utility to the survival duration of a patient from his/her health state. There was significant reduction of utility for patients experiencing AUR and having TURP procedure compared with patients at BPH state.

### Assessment Methods of Outcomes

Outcome measure is expressed as incremental cost per quality-adjusted life year (QALY) gained. As of now, there is no cost-effectiveness threshold in Hong Kong, 3 times Hong Kong GDP per capita 2017 (US$45,887) recommended by WHO-CHOICE (Evans et al., 2005) The CHOICE (CHOosing Interventions that are Cost-Effective) was used to determine if the new intervention is cost-effective compared to the existing one (Economic Analaysis Division Finance Secretary’s Office, 2018).

### Deterministic One-Way Sensitivity Analysis (OWSA)

To identify key model parameters, OWSA was conducted over the range of pre-defined values of each parameter’s point estimate (i.e., 95% confidence interval). Results were plotted in a tornado diagram according to the extent of the parameter’s impact on the incremental cost-effectiveness ratio (ICER).

### Multivariate Probabilistic Sensitivity Analysis

To assess the simultaneous influence of model parameters on the ICER, a multivariate probabilistic sensitivity analysis was performed using second-order Monte Carlo simulation (computational algorithm based on repeated random sampling of the probability distributions for each model parameter). A total of 10,000 Monte-Carlo iterations were simulated, each generating a model-estimated value for the cost and QALY. The cost-effectiveness plane was produced depicting the scatterplot of the 10,000 simulated sets of cost and QALY estimates. In addition, a cost-effectiveness acceptability curve (CEAC) was generated to display the probability of cost-effectiveness of both interventions at each willingness-to-pay threshold. **Table 2** presents the categories of model parameters, probability distributions as well as the upper and lower values used in the multivariate probabilistic sensitivity analysis.

**Table 2:** Model inputs and their distributions used in multivariate probabilistic sensitivity analysis.

Model parameter	Probability distribution	Distribution parameters	Mean/base case	LL	UL
**Probabilities**					
Probability ofBPH	Beta	α=0.62	0.0208	0.0098	0.036
patients who experience		β=29.27			
TURP					
Probability of patients	Beta	α=2.83 β=7.79	0.3295	0.275	0.41
who still remain at					
recovery state					
Probability of recovered	Beta	α=2.24	0.0743	0.0295	0.0774
patients who require		β=27.97			
medical intervention					
Probability of patients	Beta	α=2.3 β=35.16	0.0614	0.0215	0.061
on medication who					
require the second TURP					
Probability of BPH	Beta	α=0.06	0.02	0.0032	0.075
patients who experience		β=2.9			
AUR					
**Treatment effects for disease progression**					
Efficacy of	Beta	α=1.77	0.676	0.527	0.778
tamsulosin/dutasteride		β=0.85			
against AUR					
Efficacy of	Beta	α=2.5 β=1.04	0.706	0.577	0.795

tamsulosin/dutasteride					
against TURP					
**Cost**					
Managing a patient who	Gamma	α=3.61	471	223	720
is initially in BPH		λ=0.01			
moderate state					
Managing a patient who	Gamma	α=0.21 λ=1.57	1312	590	4,199
experiences an episode					
of AUR					
Managing a patient who	Gamma	α=12.59	6334	4549	8119
undergoes TURP		λ=0.002			
procedure					
Managing a patient who	Gamma	α=2.24? λ=0.006	371	123	620

requires medical					
intervention					

*LL, Lower limit; UL, Upper limit*.

The model was implemented in TreeAge pro 2017 (TreeAge Software, Williamstown, MA, United States). A microsimulation of 10,000 patients was executed to enable the tracking of the number of clinical events.

## Results

### Base-Case Analysis

As shown in **Table 3**, the Markov microsimulation based on 10,000 iterations showed that, over 4 years, compared to tamsulosin, combination therapy could prevent 0.03 AURs (∼27% reduction) per patient, 0.07 TURPs (∼32% reduction) per patient, and 0.002 deaths (∼3.5% reduction) over 4 years. Compared to tamsulosin, combination therapy can lead to cost reduction of US$31, US$1,029, and US$2 per patient due to reduced episodes of AUR, TURP and need of medical intervention, respectively and additionally a delay in patient’s progression to AUR and TURP. However, those benefits came with an incremental drug cost of US$1,543 per patient over 4-year period. The increased number of BPH (3.43) for combination therapy compared to that (3.3) of monotherapy was mainly due to the delayed progression of BPH patients to AUR and TURP health states. In addition, the analysis showed that a patient on combination therapy would experience more QALYs (3 vs. 2.93 QALYs) than those on monotherapy. As a result, replacing monotherapy by combination therapy was expected to lead to US$11,651 per QALY gained.

**Table 3:** Base-case results.

	Combination therapy (a)	Monotherapy (b)	Difference (a-b)
*Model time horizon: 4 years*			
**Average cost per patient of 10,000 population (US$ 2018)**			
Drug	1,749	206	1,543
BPH	1,935	1,642	293
AUR	108	139	-31
TURP	1,090	2,119	-1,029
Medical intervention	6	8	-2
**Health outcomes per patient (by episode)**			
BPH	3.43	3.3	0.15
AUR	0.08	0.11	-0.03
TURP	0.22	0.29	-0.07
Death	0.056	0.0577	-0.002
QALY	3.00	2.93	0.07
**Incremental cost-effectiveness ratio**			
Cost per QALY gained	11,651		
*Model time horizon: Life-time (35 years)*			
**Average cost per patient of 10,000 population (US$ 2018)**			
Drug	7,473	856	6,617
BPH	5,224	4,475	749
AUR	2,644	3,095	-451
TURP	6,124	11,594	-5,470
Medical intervention	212	249	-37
**Health outcomes per patient (by episode)**			
BPH	11.09	9.5	1.59
AUR	2.01	2.36	-0.35
TURP	2.25	2.62	-0.37
Death	0.9675	0.9784	0.0109
QALY	10.29	9.87	0.42
**Incremental cost-effectiveness ratio**			
Cost per QALY gained	3,329		

As the modeling time horizon was extended to life-time, compared to monotherapy, more health benefits were projected for patients receiving combination therapy, i.e., a reduction of 0.35 AURs per patient, 0.37 TURPs per patient and 0.0109 deaths, which translated into 0.42 QALY gained per patient. In addition, using combination therapy at an incremental drug cost of US$6,617 compared to tamsulosin could potentially save US$451 per patient in managing AUR and US$5,470 per patient in TURP procedure. Similarly, as the combination therapy reduced risks of a patient progressing to AUR and TURP health states, therefore, an incremental cost of US$749 in managing BPH was incurred.

#### Way Sensitivity Analysis

**FIGURE 2:**
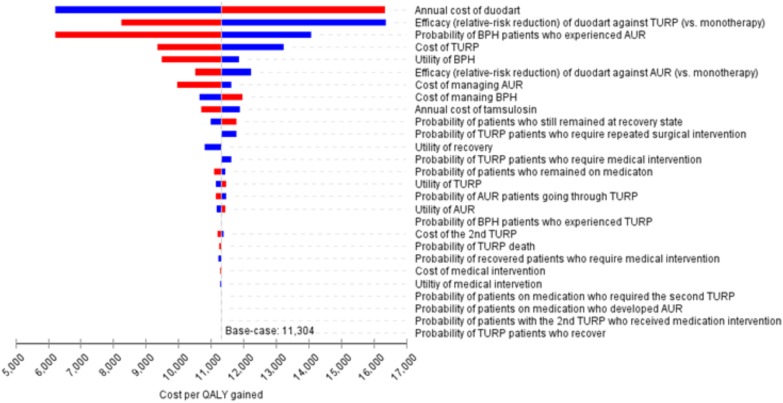
Tornado diagram for one-way sensitivity analysis. ^#^The results were based on 4-year projection.

The OWSA showed that the top five most influential parameters on ICER were annual cost of tamsulosin/dutasteride, efficacy of tamsulosin/dutasteride against TURP, and probability of BPH patients who experienced AUR, cost of providing TURP procedure and utility of BPH. The remaining parameters had only a moderate influence on ICER estimates causing ICER to vary by no more than US$2,000. On the whole, all the ICERs were found to be between US$6,000 and US$17,000 per QALY when parameters were varied over their uncertainty range (**Figure 2**).

### Multivariate Probabilistic Sensitivity Analysis

**FIGURE 3:**
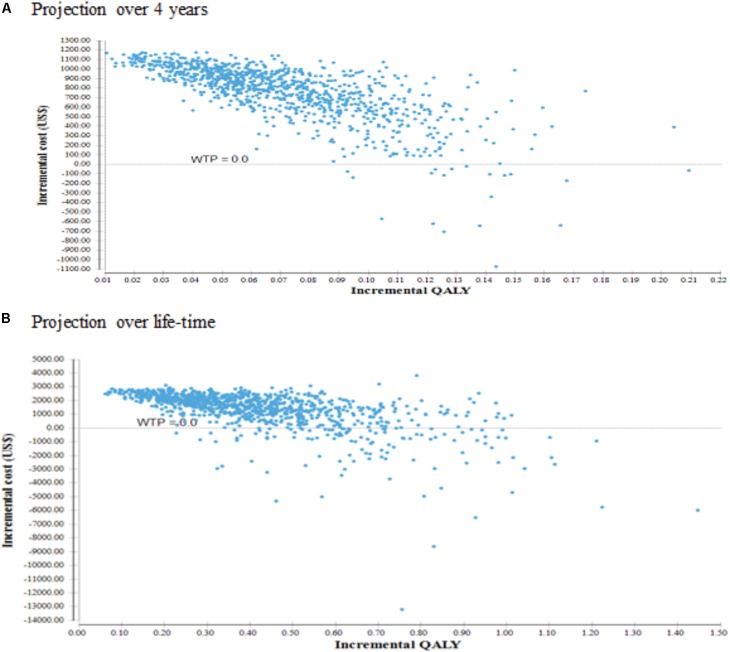
Cost-effectiveness scatterplot. **(A)** Cost effectiveness projection over 4 years. **(B)** Cost effectiveness projection over life-time.

The average incremental costs and QALYs over the 10,000 Monte Carlo simulations from the probabilistic sensitivity analysis were essentially similar compared to the deterministic base-case results. As shown in **Figure 3A**, over 4-year projection period, the scatterplots of incremental costs and QALYs for combination therapy vs. monotherapy showed that over 98% of the 10,000 Monte Carlo iterations fell within the north-east quadrant, suggesting that combination therapy produced higher QALYs at a higher cost, even with uncertainty in all parameters. In **Figure 3B**, over life-time horizon, there was a trend suggesting that combination therapy was cost-saving, i.e., less costly and more effective, among 12.8% of all 10,000 simulations while the remaining simulated results fell within the north-east quadrant.

**FIGURE 4:**
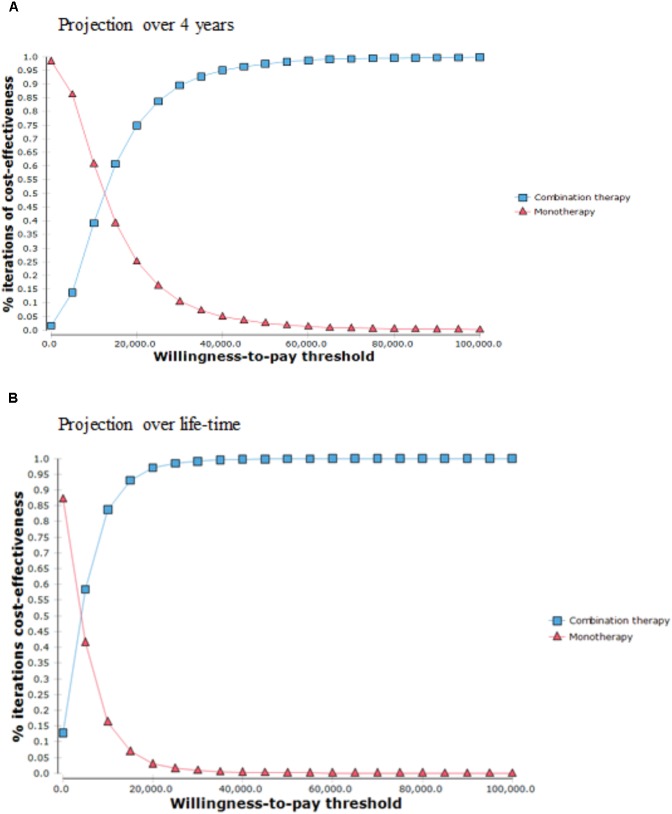
Cost effectiveness acceptability curve. **(A)** Projection over a 4 year period. **(B)** Projection over lifetime period.

As shown in **Figure 4**, the CEAC showed that, over 4-year time horizon, it demonstrated that the probability of combination therapy being cost-effective was greater than 95% if willingness to pay (WTP) threshold is above US$40,000. Besides, the combination therapy had a probability of 97% of being cost-effective over monotherapy at one time HK GDP per capita of US$45,887. Over life-time projection, combination therapy would have a probability of cost-effectiveness of more than 95% if WTP threshold is above US$20,000. It will achieve a probability of 99% being cost-effective at one time HK GDP per capita.

## Discussion

Hong Kong’s healthcare system comprises of the public and private sector, with the former being the cornerstone of the healthcare system, acting as a safety net for the population. The public system currently serves 90% of all inpatient needs and ∼30% of outpatient services (Kong et al., 2015). It has a very comprehensive range of services which are charged at very low rates, representing approximately 95% subsidy from the actual cost. Similar to many countries around the world, Hong Kong is currently facing serious challenges due to its rapidly aging population (Kong et al., 2015; Lee and Mak, 2016; Ang et al., 2017). It is estimated that the total population aged >60 years in Hong Kong will rise from 12.5% currently to 25% in 2030, which will thus result in an increase in BPH prevalence. 5ARIs and alpha blockers, used either as monotherapy or combination therapy, are common modalities for BPH/LUTS treatment. While the results of the CombAT trial proved the clinical benefits of dutasteride/tamsulosin combination in symptom relief and reducing clinical progression of BPH (Roehrborn et al., 2010), our model based on current drug price showed that patients who were treated with combination therapy experienced more QALYs but incurred higher costs than patients on background medications, leading to an ICER of US$11,651 and US$3,329 per QALY over 4-year and life-time horizon. Deterministic OWSA indicated that the results were most sensitive to the acquisition cost of combination therapy and drug efficacy.

It is worth noting that based on hospital data 80% of all patients with AUR would eventually go through the most common surgical procedure for BPH treatment, i.e., TURP. This is reflective of the reality as a small number of patients would not be fit for surgery due to various medical reasons, and besides TURP there are other surgical options for the management of TURP, e.g., laser prostatectomy. However, in the setting of the local health care system, TURP is still the majority. Such assumption for the construction of the Markov model would give a good estimation of the results.

Despite the differences in epidemiology and costs, our findings were consistent with the other published cost-effectiveness analyses which also showed that combination therapy led to the potential incremental cost and benefit of QALYs gained, leading to cost-effectiveness of the new intervention. For example, a study in Canady by Ismaila et al. (2013) showed that, over a lifetime, the combination therapy led to an ICER of US$20,224 per QALY gained over 10-year time horizon and was considered cost-effective for 99.6% of probability at a willingness-to-pay threshold of CAN$50,000 (US$39,752) per QALY. In the United Kingdom, Walker et al. (2013) constructed a Markov state transition model measured by QALYs for patients aged ≥50 years with BPH and moderate to severe symptoms. While cumulative discounted costs per patient were higher with combination therapy than with tamsulosin alone, QALYs were also higher. Their probabilistic sensitivity analysis revealed that the probability of combination therapy being cost-effective lied in the range of 78–88%. In a Norwegian economic model, Bjerklund Johansen et al. (2012) used a semi-Markov model to project costs and utility outcomes over two time horizons, namely 4 years and lifetime. ICERs of combination were US$12,374 and US$6,871 over 4 years and lifetime, respectively. Another Japanese study on the pharmacoeconomic evaluation of combination therapy suggested that such treatment with alpha blocker and dutasteride in BPH patients would be more cost-effective than alpha blockers alone in patients with moderate to severe symptoms (Takayama et al., 2012). The ICERs for combination therapy versus monotherapy calculated at 4 years and 10 years were US$47,581 and US$55,532/QALY gained, respectively, both below the acceptable ICER threshold in Japan.

The present study has several limitations. Firstly, in our Markov model, due to data availability, we did not separate the state of symptomatic BPH into patients with mild, moderate and severe symptoms according to IPSS score. Instead, these 3 groups of patients were analyzed as a single group. Such categorization might have made the prediction of AUR probability less precise. As shown in OWSA, the utility of BPH has substantial impact on ICER. Future study on classification of BPH severity was thus warranted. Secondly, we didn’t model the occurrence of adverse events because, as reported in the ComBAT trial, the incidences of adverse events were very similar between combination therapy and monotherapy. In a 2015 study, complete absence of ejaculation was experienced by 23% of patients on combination therapy, and 15% on tamsulosin alone (Stojanoviæ et al., 2015). In the same study, it was found that erection improved in both groups of the patients. Overall, the difference in adverse events between the 2 groups is very small and is expected to have minimal impact on the cost-effectiveness results. Thirdly, we didn’t provide analysis from societal perspective which includes indirect costs associated with BPH patients. Indirect costs associated with BPH would include cost from complications of BPH e.g., urinary tract infection, bladder stones, and hematuria (Leong et al., 2014; Lee et al., 2015). It also would include the loss of productivity of patients and caregivers. The inclusion of those costs is expected to make the cost-effective results even more favorable. Finally, our model were based upon data from literature which dates back to 1990s. While this may not be truly representative of the current situation, we had to rely on such classical studies for the input of the model. Nevertheless, we had also sought experts input to ensure that the model was representative of the current situation in Hong Kong to ensure its applicability.

In conclusion, our study shows that combination therapy as compared with monotherapy is cost-effective due to substantial reduction in the number of AUR and TURP and the associated direct cost in BPH patients in Hong Kong.

## Data Availability

Data sharing is not applicable to this article as no datasets were generated or analyzed during the current study.

## Author Contributions

DW played a principal role in the study concept and design, performed the statistical analysis, interpreted the results, and wrote the draft. CY designed the study, provided statistical support, reviewed, and edited the manuscript. C-FN, KL, SL, and NC contributed to data acquisition or interpretation of data. Y-SC provided inputs in reviewing the methodology part and assisted with model calibration using TreeAge software. KL obtained the funding. All authors approved the final manuscript.

## Conflict of Interest Statement

The authors declare that the research was conducted in the absence of any commercial or financial relationships that could be construed as a potential conflict of interest.
